# Case of Inversion of the Vagina Coming on during Labor

**Published:** 1851-11

**Authors:** 


*Case of Inversion of the Vagina coming on during Labor.* By Dr. LAMBERT.—The patient was a laboring woman. During the last six months of her third pregnancy she suffered from prolapsus of the vagina whenever she was working at out-of-door labor. The swelling thus produced attracted her attention, but did not alarm her, as it disappeared when she lay down in bed.

When labor came on, the tumor again appeared between the limbs. The midwife in attendance finding the labor tedious, and ignorant of the nature of the case, recommended the woman to make the most of her pains, and ordered her a vapor bath. The only apparent effect of this advice was the increase of the tumor to double its former size. When Dr. Lambert arrived, he found it projecting from the vulva, of the size of the two fists, of a blueish-red color, round, wrinkled, and of considerable consistence. At its lower extremity was an opening through which the finger could be introduced to the os uteri.

Dr. L. recommended rest in the horizontal posture, with the pelvis a little elevated, cold applications to be made to the tumor, and slight pressure applied to replace it during the intervals between the pains. After considerable delay the tumor was reduced, and the woman delivered with the forceps. She made a good recovery, and the swelling has never returned.—*London Monthly Journal from Revue Med. Chir.*, April 1851.

